# Use of Prognostication Instruments in Prognostication Procedures of Postanoxic Coma Patients over Time: A Retrospective Study

**DOI:** 10.3390/jcm12103357

**Published:** 2023-05-09

**Authors:** Charlotte Daun, Anne Ebert, Vesile Sandikci, Simone Britsch, Kristina Szabo, Angelika Alonso

**Affiliations:** 1 Department of Neurology, Mannheim Center for Translational Neuroscience, Medical Faculty Mannheim, University of Heidelberg, 68167 Mannheim, Germany; 2 Department of Cardiology, Medical Faculty Mannheim, University of Heidelberg, 68167 Mannheim, Germany

**Keywords:** cardiac arrest, coma, hypoxic ischemic encephalopathy, prognosis

## Abstract

Background: Many survivors of cardiovascular arrest remain in a postanoxic coma. The neurologist’s task is to provide the most accurate assessment of the patient’s neurologic prognosis through a multimodal approach of clinical and technical tests. The aim of this study is to analyze differences and developments in the concept of neurological prognosis assessment and in-hospital outcome of patients over a five year-period. Methods: This retrospective observational study included 227 patients with postanoxic coma treated in the medical intensive care unit of the University Hospital, Mannheim from January 2016 to May 2021. We retrospectively analyzed patient characteristics, post-cardiac arrest care, and the use of clinical and technical tests for neurological prognosis assessment and patient outcome. Results: Over the observation period, 215 patients received a completed neurological prognosis assessment. Regarding the multimodal prognostic assessment, patients with poor prognosis (54%) received significantly fewer diagnostic modalities than patients with very likely poor (20.5%), indeterminate (24.2%), or good prognosis (1.4%; *p* = 0.001). The update of the DGN guidelines in 2017 had no effect on the number of performed prognostic parameters per patient. The finding of bilaterally absent pupillary light reflexes or severe anoxic injury on CT contributed most to a poor prognosis category (OR 8.38, 95%CI 4.01–7.51 and 12.93, 95%CI 5.55–30.13, respectively), whereas a malignant EEG pattern and NSE > 90 µg/L at 72 h resulted in the lowest OR (5.11, 95%CI 2.32–11.25, and 5.89, 95%CI 3.14–11.06, respectively) for a poor prognosis category. Assessment of baseline NSE significantly increased over the years (OR 1.76, 95%CI 1.4–2.22, *p* < 0.001), and assessment of follow-up NSE at 72 h trended to increase (OR 1.19, 95%CI 0.99–1.43, *p* = 0.06). In-hospital mortality was high (82.8%), remained unchanged over the observation period, and corresponded to the number of patients in whom life-sustaining measures were discontinued. Conclusions: Among comatose survivors of cardiac arrest, the prognosis remains poor. Prognostication of a poor outcome led nearly exclusively to withdrawal of care. Prognostic modalities varied considerably with regard to their contribution to a poor prognosis category. Increasing enforcement of a standardized prognosis assessment and standardized evaluation of diagnostic modalities are needed to avoid false–positive prognostication of poor outcomes.

## 1. Introduction

Postanoxic coma is a correlate of global cerebral ischemia, resulting from cardiovascular arrest and the associated oxygen deficiency in the brain [1]. In Europe, the incidence of out-of-hospital cardiovascular arrests in adults ranges between 67 and 170 per 100,000 inhabitants [2], and sudden cardiac death due to cardiovascular arrest is the third leading cause of death in Europe [3].

Due to advanced resuscitation techniques, more and more patients achieve a return of spontaneous circulation after resuscitation in the context of a cardiovascular arrest, but the survival rate of patients at hospital discharge remains low, averaging at about 30% of hospital-admitted patients [3,4]. 

The chance of comatose survivors of cardiac arrest to regain consciousness gradually decreases over time in coma [5], and up to 30% of surviving patients may finally be in a syndrome of unresponsive wakefulness if life-sustaining measures are continued [6].

The management of patients with postanoxic coma requires an interdisciplinary cooperation of neurologists and intensive care physicians. The demand on the neurologist is accompanied by an enormous expectation to make early and accurate predictions about long-term prognoses [7]. Discontinuation of life-sustaining measures based on neurologic prognostic assessment is the main cause of death in patients with postanoxic coma [8,9]. As a result, neurologic prognostic assessment significantly influences the patient’s therapy.

Various clinical parameters, neurophysiological tests, and biomarkers can be used to assess the prognosis of postanoxic coma. However, each factor alone has a limited specificity and thus quickly implies a poor prognosis that may lead to treatment discontinuation [10,11]. In addition, there is currently no parameter available that can predict the neurologic prognosis after cardiovascular arrest without limitations [12]. Furthermore, the timing of neurological assessment plays a major role. 

This retrospective study analyzes differences and trends over time regarding the post-cardiac arrest care, the neurological prognostic assessment, and the in-hospital outcome of the patients during the time of observation from January 2016 to May 2021.

## 2. Methods

We retrospectively analyzed 227 patients treated at the Mannheim University Hospital between January 2016 and May 2021. To avoid selection bias, the hospital database management system was screened for all patients over 18 years who were resuscitated medically and/or mechanically in-hospital or out-of-hospital by ICD-10 codes (I46.0, U46.1, I46.9). Patients were included if (1) resuscitation was successful with return of spontaneous circulation, (2) they were diagnosed with postanoxic coma > 24 h after resuscitation, and (3) a neurological consultation was performed >/= 72 h after resuscitation. All patients were treated in the medical intensive care unit of the Mannheim University Hospital. 

The retrospective data collection was performed using the electronic and paper records of Mannheim University Hospital. We analyzed basic demographic information, such as age, sex, length of stay, as well as the prevalence of pre-existing conditions and the premorbid modified Rankin Scale (mRS). In addition, we analyzed whether patients received targeted temperature management (TTM). According to local standards, TTM was induced by rapid infusion of cold intravenous fluids (20–30 mL/kg) and continued via intravascular cooling catheter. The target temperature of 33 °C had to be reached within 4 h of reperfusion and was maintained for 24 h, followed by controlled rewarming at 0.3 °C per hour. Neuron-specific enolase (NSE) values and neurologic examination findings (brain stem reflexes, Glasgow coma scale motor score) performed by neurological consultants at least 72 h after cardiopulmonary resuscitation were recorded. Evaluation of CT scans in our cohort was performed by experienced neuroradiologists by qualitative assessment of diffuse cerebral edema, effacement of the junction between gray and white matter, and diffuse hypodensity of cerebral parenchyma. Evaluation of electroencephalography (EEG) and somatosensory evoked potentials (SSEP) was performed by the neurological consultant and verified by a senior neurologist. According to the guideline recommendations, pupillary light response and EEG were only turned into account in patients with no or minimal sedation. Neurological prognosis was categorized into poor (defined as syndrome of unresponsive wakefulness or death), very likely poor, indeterminate, and good according to the guideline recommendations of the German Society of Neurology (Deutsche Gesellschaft für Neurologie, DGN) in 2017 [13]. 

Withdrawal of treatment was recommended to legal representatives by neurological consultants in case of poor prognosis. In patients with very likely poor prognosis, the possibility of withdrawal of treatment was discussed by neurological consultants with the legal representatives taking into account the patient’s (putative) will. Discharge mode was labeled as deceased, discharge to rehabilitation hospital, or discharge to home care. Patients discharged alive were categorized into a Cerebral Performance Categories Scale (CPC) score of 4 (syndrome of unresponsive wakefulness) and better than 4. Assessment of CPC scores was performed by neurological consultants.

### Statistical Analysis

Statistical analysis was performed using IBM SPSS^®^ Statistics 27.0, IBM North America, New York, NY, USA. Absolute and relative frequencies were reported for categorical variables, such as gender, the premorbid modified Rankin scale, the use of TTM, and preexisting conditions. Likewise, absolute and relative frequencies were reported for the number of performed prognostic tests (SSEP, EEG, CT/MRI, NSE), neurologic prognosis, and patient discharge mode. For metric variables, such as age and length of stay, mean and standard deviation, or, in the case of considerable variation, median and interquartile range (IQR) were reported. The sum of performed prognostic tests per patients was compared between patients with poor versus other outcome category, as well as in the years prior to the 2017 DGN guideline update (published online 03/2018) versus after the update using Mann Whitney U test. Odds ratios for increasing risk of poor prognosis by negative outcome predictor were calculated, as well as Chi^2^-tests. Logistic regression was used to analyse changes over time in the frequency of TTM, diagnostic modalities, and outcome. For this purpose, use of TTM, SSEP, EEG, CT, NSE and neurological examination (pupillary light reflex), prognosis category “poor”, and death were each defined as dependent variables, and the year of performance was defined as the independent variable. We refrained from logistic regressions with the remaining prognosis categories due to low absolute numbers. Results are reported as Exp (B), indicating the Odds Ratio and *p*-value. A *p*-value of <0.05 was considered significant.

## 3. Results

### 3.1. Demographic Information and Preexisting Conditions

The present study includes 227 patients who were treated in the medical intensive care unit of the University Medical Center Mannheim within the observation period from 1 January 2016 to 5 February 2021. The mean age was 66.3 ± 14.7 years, 155 Patients (68.3%) were male, and length of stay varied considerably, with a median of 10 days (IQR 7–19, range 1–112). On average, 42 patients with postanoxic coma were included per year. Most frequent comorbidities were end-stage renal failure or dialysis (68 patients; 30.0%), chronic lung disease (50 patients; 22.0%), and heart failure ≥ NYHA III (44 patients; 19.4%). Over the years, there was a significant increase up to 58.8% of patients in 2021 who received TTM after return of spontaneous circulation (*p* = 0.001, Exp (B) 1.39, 95%CI 1.14–1.70, indicating a 1.39-fold increase in TTM per year). Table 1 summarizes baseline characteristics of the cohort.

### 3.2. Modalities for Neurological Prognosis

The most frequent modality for neurological prognostication over the years was computed tomography, which was requested by the neurological consultant in 177/227 (78%) of cases. In contrast, magnetic resonance tomography had little use in neurologic prognostic assessment: only 3.4% of patients diagnosed with postanoxic coma received magnetic resonance tomography. A clinical examination with documented pupillary light reaction was performed in 172/277 (75.8) patients in a time span of at least 72 h after resuscitation. Somatosensory evoked potentials (SSEP) were performed in 156/227 (68.7%) patients, and electroencephalography (EEG) was performed in 169/227 (74.4%) patients. Despite some variation in the frequency of these modalities between single years, logistic regression did not show a significant trend over time for the frequency of CT, neurological examination, SSEP, or EEG (see Table 2). In contrast, analysis of baseline NSE at 24 h showed a significant correlation with time (*p* < 0.001), resulting in a 1.76-fold likelihood per advancing year (95%CI 1.4–2.22) and a frequency of 163/277 (71.8%). NSE at 72 h, however, was measured less frequently (mean 125/277, 55.1%), and a trend towards increasing determination rates over the years, with an odds ratio of 1.19 (95%CI 0.99–1.43), missed statistical significance (*p* = 0.06). Table 2 gives an overview of application frequencies of prognostication instruments over the years.

### 3.3. Neurological Prognosis and Outcome over the Years

An amount of 68/156 (43.6%) patients examined with SSEP showed bilaterally absent cortical potentials. An amount of 122/169 (72.2%) patients receiving an EEG had a “malignant” EEG pattern. A burst suppression pattern was diagnosed in 10 patients, suppressed EEG was diagnosed in 86 patients, and continuous generalized spike wave discharges with a frequency ≥ 3/s were diagnosed in 26 patients. Cranial CT revealed severe anoxic injury in 59/177 (33.3%) patients and mild signs of postanoxic injury in 55/177 patients. Brain death was confirmed by CT/CT-angiography in two patients. On MRI, mild signs of postanoxic injury could be diagnosed in 6/9 patients.

Over the observation period, 215 patients received a completed neurological prognosis assessment during their hospitalization, and 12 patients died before prognosis completion. The prognosis was judged as poor in 116/215 patients (54%), very likely poor in 44/215 patients (20.5%), indeterminate in 52/215 patients (24.2%), and good in 3/215 patients (1.4%, see Table 3). 

The number of performed prognostic parameters was significantly lower in patients with poor prognosis (median 4, IQR 3–5 versus median 5, IQR 4–5; *p* = 0.001), whereas the 2017 update of the DGN guidelines had no effect on the number of performed prognostic parameters per patient (median pre-update 5, IQR 4–5 versus median post-update 4, IQR 3–5; *p* = 0.14).

**Figure 1:**
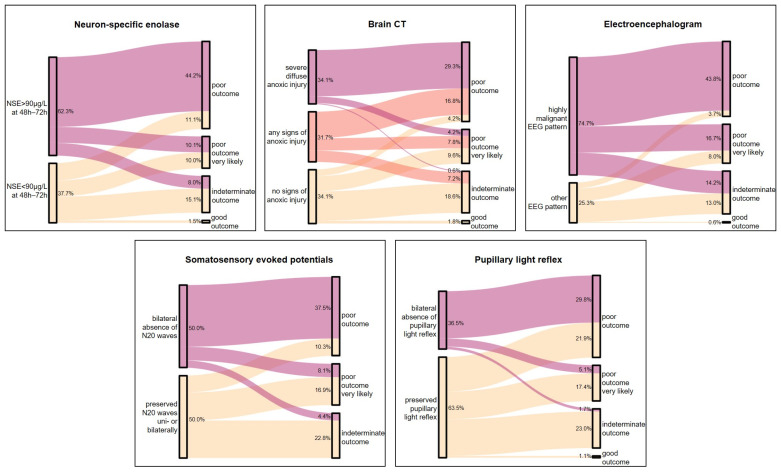
Sankey Plots indicate the contribution of each diagnostic modality to the prognosis category.

Figure 1 shows the contribution of each prognostic parameter to the final prognosis category. 

The finding of bilaterally absent pupillary light reflexes or severe anoxic injury on CT contributed most to a poor prognosis category (OR 8.38, 95%CI 4.01–17.51 and 12.93, 95%CI 5.55–30.13, respectively), whereas a malignant EEG pattern and NSE > 90 µg/L at 72 h resulted in the lowest OR (5.11, 95%CI 2.32–11.25, and 5.89, 95%CI 3.14–11.06, respectively) for a poor prognosis category. In the case of bilaterally absent N20 potentials in SSEP, the OR for a poor outcome category was 6.56 (95%CI 3.21–13.38).

Regarding short-term outcome, all patients diagnosed to have poor outcome deceased during hospitalization. Death occurred as a consequence of withdrawal of care in 113/116 patients. Of 44/215 patients in whom prognosis was rated as very likely poor, 39 patients deceased during hospitalization, and 38 were due to withdrawal of care. Five patients were transferred to rehabilitation or home care. Of these, four patients had a Cerebral Performance Categories Scale score (CPC) of 4, and one patient had a CPC of <4. Twenty-one patients (24.2%) with a prognosis graded as indeterminate (52/215) were deceased. A withdrawal of care was applied in 19/21 patients. An amount of 31 patients were transferred to a rehabilitation facility or nursing home care. Of these, eight patients had a CPC of 4 and 23 patients had a CPC < 4. All three patients with a good neurological prognosis were transferred to a rehabilitation facility or care at home and no limitation of therapy was agreed for any of them. All patients had a CPC < 4 at discharge. 

Over the observation period, logistic regression identified a trend towards an increase in the rate of prognoses graded as poor, with a 1.20-fold increase per year (95%CI 0.99–1.45) (*p* = 0.06). In comparison, the rate of patients dying with a diagnosis of postanoxic coma stayed the same, without significant trends to increase or decrease over the period (*p* = 0.24, Exp (B) 1.16, 95%CI 0.91–1.47, see Table 3).

## 4. Discussion

Main findings of our analysis are (1) prognosis of comatose survivors of cardiac arrest was mostly poor, (2) there was substantial variability in frequency and number of prognostic parameters performed, as well as in their contribution to the final prognosis category, and (3) in-hospital mortality was high and almost exclusively occurred due to withdrawal of care. 

### 4.1. Outcome Prognostication

In line with previous studies [6,14], the rate of patients with poor prognosticated outcome was very high. We noted a trend towards an increase in patients with a poor prognosis over time. The concept of a multimodal prognostication strategy algorithm has been proposed by the European Resuscitation Council (ERC)/European Society of Intensive Care Medicine (ESICM) in 2015, aiming at a minimal false positive rate when predicting poor outcome [15]. With some variations, the concept has been adopted by the 2017 guideline of the German Society of Neurology (DGN) [13]. The algorithm includes an overall assessment of congruent results from at least three clinical and technical tests. A multimodal diagnostic assessment is applied after the use of a TTM at 32–34 degrees over 24 h and a subsequent rewarming. This includes a neurological examination, preferably without sedation or muscle relaxation [13]. Absence of pupillary response, NSE > 90 µg/L, bilateral absence of N20 potential, highly malignant EEG, and severe signs of postanoxic injury on CT or MRI are classified as parameters of poor neurologic prognosis. If ≥ 3 of the parameters are positive, a poor prognosis may be determined; in this case, a withdrawal of life-sustaining measures is now recommended. In the case of one or two positive parameters, the prognosis is most likely poor. Without clinical improvement on day 7 and an indication of severe hypoxic brain injury in a repeat SSEP, EEG, CT, or MRI scan, limitation of therapy should also be discussed. 

### 4.2. Prognostic Parameters

We found that the number of performed prognostic parameters per patient was significantly lower in patients with poor outcome prognostication. The current DGN guidelines allow the prognosis of a poor outcome based on three clinical and technical tests. As a consequence, performance of three clinical or technical tests is sufficient if all parameters are congruent, and performance of more or all prognostic parameters is only necessary in indeterminate cases. However, the contribution of the different negative outcome parameters to the final prognosis differed considerably in our study. Interestingly, a “malignant” EEG pattern was associated with a poor prognosis category in only 58.6% of patients. Most importantly, we did not assess the association of prognostic parameters with the true outcome, but with the prognostic category. Still, given a high positive predictive value of an algorithm-base prognosis category “poor” with true outcome [16], the diagnosis of a “malignant” EEG pattern might have a low specificity in our study. Of interest, a low-voltage EEG background (<10 µV) was not included in the 2015 ERC/ESICM guideline algorithm, as amplitude of the EEG signal may depend on several confounding factors, such as drugs, body temperature, and technical conditions [15]. In contrast, the updated 2021 ERC/ESICM guideline classify any suppressed background EEG (<10 µV) as highly malignant EEG pattern [17]. Still, evaluation of EEG is highly dependent on expert knowledge. Whereas, interrater variability for a malignant EEG pattern in post cardiac arrest comatose patients was found to be very low between senior neurophysiologists, and the Kappa score was only fair or slight between the junior and senior neurophysiologists [18]. Diagnostic criteria, as proposed by the American Clinical Neurophysiology Society [19], might help to reduce interrater variability [20]. Moreover, complex EEG patterns should be counterchecked by a senior neurophysiologist. In the future, automated tools for EEG pattern evaluation may additionally increase the specificity of EEG diagnosis.

Cranial CT was the most often performed diagnostic modality in our study, and the diagnosis of severe anoxic injury contributed most to the final prognostication category “poor”. Imaging analyses were conducted according to the current DGN guideline. A pattern of severe anoxic injury was defined as a distinct brain edema with complete effacement of the junction of gray and white matter [13]. Although the guideline provides a suggested cut-off for the gray/white matter ratio of 1.0, a quantitative analysis is not clearly recommended over a qualitative assessment of established morphological signs. Results from a recent retrospective CT imaging study in 91 patients with postanoxic coma indicate a higher interrater agreement of quantitative compared to qualitative analysis of the gray–white matter ratio [21]. However, another study found clinically relevant deviations of quantitative gray–white matter ratio measurement in individual patients, and the authors conclude that additional qualitative analyses are needed to verify the quantitative assessments [22]. Moreover, the quantitative analysis bears some intrinsic caveats that are discussed in the 2021 ERC/ESICM guideline [17]: as gray–white matter ratio is a continuous variable, a threshold to dichotomize into positive and negative results has to be defined. Sensitivity and specificity will vary depending on the chosen cut-off value. The recommended cut-off value of 1 according to the DGN guidelines will thus yield high specificity at only moderate sensitivity, and previous studies have applied various cut-off values, mostly ranging from 1.1 to 1.2 [22,23]. Moreover, there is no consensus recommendation on calculation of the gray–white matter ratio with regard to number, location, and volume of defined regions of interest. To establish a valid tool for quantitative CT evaluation, obligatory methodological standards need to be defined. 

### 4.3. In Hospital Outcome

In line with previous data [24,25], death due to neurological injury was rare, but it occurred nearly exclusively as a consequence of withdrawal of care. Life-sustaining measures were discontinued in most to nearly all patients with a poor or very likely poor neurologic prognosis, but also in a relevant number of patients with indeterminate prognosis. According to the DGN guidelines, withdrawal of care was recommended in patients with poor prognosis and discussed in patients with very likely poor prognosis, taking into account the putative patient’s will. Consequently, the high mortality in patients with poor or very likely poor prognosis in our cohort is explained and justified by a consistent implementation of guideline recommendations. In the literature, single case reports of patients who, despite a poor neurologic prognosis, nevertheless experienced favorable recovery within one year if intensive measures were continued, can be found [26]. In this context, it would be interesting to re-evaluate the respective prognostication assessments to exclude false–positive interpretations, as a large study evaluating the ERC/ESICM prognostication algorithm yielded a false-positive rate of 0% if any combination of two modalities indicated a poor prognosis [16].

Early limitation of treatment in patients with indeterminate prognosis as implemented in about one third of this prognostic subgroup in our cohort, however, should be appraised with caution. It is possible that premature prognosis and resulting erroneous and unilateral decisions regarding therapy discontinuation in the treatment of a postanoxic coma are likely to bias patient treatment attempts and survival rates toward failure [27]. Additionally, in this context, the sedation strategy, along with the use of a TTM, should be noted. Late awakening after cardiovascular arrest with appropriate drug sedation and the use of a TTM is not always associated with poor neurological outcome [28]. Although we have no data regarding the process of decision-making in individual patients, it is likely that the majority of patients with indeterminate prognosis were (still) in a nonresponsive state at the time of withdrawal of treatment. Thus, caregivers might anticipate an unfavorable outcome at the time of decision-making as the lack of consciousness is mostly perceived as even worse than death [29]. Neurological consultants or intensive care physicians should therefore be trained in counselling caregivers of postanoxic coma patients. The possibility of recovery over several months has to be explained, but also the option of a change in treatment goals in case of absent recovery in a reasonable time frame. Of course, we cannot exclude that other causes, such as premorbid status and concomitant diseases, led to a withdrawal of care in patients with indeterminate prognosis. No patient with a good neurologic prognosis received therapy limitation in our study. In four of five patients who were discharged with an indeterminate or good neurological prognosis, the CPC was better than four at the time of discharge. Although we have no data on functional outcome at three or six months, we can extrapolate that the rate of long-term survivors with very poor functional outcome, defined as CPC 4–5, will thus be low. In countries where withdrawal of life supporting therapy (WLST) is not practiced, surviving patients are more likely to have a worse neurologic outcome [2,30]. The support of WLST is highly dependent on cultural aspects, underlying ethical conceptions and values. Approval of WLST has been shown to be associated with endorsement of the importance of patient autonomy, the presence of consciousness, as well as the ability to interact with others [31].

### 4.4. Limitations

Due to the retrospective nature of the study, the quality of data rests on the quality of existing documentation. The sample size is moderate, but the cohort is very homogeneous and well characterized. Data on long-term outcomes are not available; however, the aim of the study was to characterize procedural aspects, not accuracy of neuroprognostication. The interpretation of diagnostic parameters and prognostic categories were recorded, as determined by the treating neurologist; diagnostic tests and prognosis category were not re-evaluated by the authors. Thus, we cannot draw any conclusions regarding interrater variability and accuracy of diagnostic interpretation.

## 5. Conclusions

In summary, we could corroborate previous data regarding poor prognosis of comatose cardiac arrest survivors. Avoidance of false-positive prognosis of poor outcome is crucial to minimize self-fulfilling prophecies. However, prognostic modalities varied considerably with regard to their contribution to a poor prognosis category. This finding underlines the need for explicit criteria for the interpretation of each diagnostic modality in this setting. Moreover, prognostication should be supervised by experienced clinicians and neurophysiologists.

## Figures and Tables

**Table 1:** Patient Characteristics (*n* = 227).

**Age, Median (SD), Years**	66.3 (14.7)
Length of stay, median (SD), days	15.7 (15.9)
Sex	
Male, *n* (%)	155 (68.3)
Female, *n* (%)	72 (31.7)
Premorbid modified Rankin Scale	
0, *n* (%)	87 (38.3)
1, *n* (%)	40 (17.6)
2, *n* (%)	37 (16.3)
3, *n* (%)	38 (16.7)
4, *n* (%)	20 (8.8)
5, *n* (%)	5 (2.2)
Pre existing condition	
Chronic kidney disease/Dialysis, *n* (%)	68 (30.0)
Chronic pulmonary disease, *n* (%)	50 (22.0)
Heart failure ≥ NYHA III, *n* (%)	44 (19.4)
Active cancer diagnosis, *n* (%)	34 (15.0)
Dementia, *n* (%)	11 (4.8)
Liver cirrhosis Child-Pugh Score C, *n* (%)	5 (2.2)
Life expectancy < one year, *n* (%)	5 (2.2)
Pathogenesis of hypoxic-ischemic injury	
Cardiac arrest, *n* (%)	221 (97.4)
Respiratory failure, *n* (%)	84 (38.0)
Arrhythmia without ACS, *n* (%)	45 (20.4)
ACS, *n* (%)	39 (17.6)
Unknown causes, *n* (%)	27 (12.2)
Non-cardiogenic shock, *n* (%)	13 (5.9)
Other causes, *n* (%)	13 (5.9)
Primary hypoxic, preserved CBF, *n* (%)	6 (2.6)
Arrest Location	
Out-of-hospital, *n* (%)	161 (70.9)
In-hospital, *n* (%)	66 (29.1)
Bystander CPR, *n* (%)	88 (38.8)
First monitored rhythm	
Asystole, *n* (%)	101 (44.5)
Pulseless electrical activity, *n* (%)	54 (23.8)
Ventricular fibrillation, *n* (%)	51 (22.5)
Unknown, *n* (%)	21 (9.2)
Targeted Temperature Management	
2016, *n* (%)	9 (39.1)
2017, *n* (%)	10 (29.4)
2018, *n* (%)	9 (15.3)
2019, *n* (%)	14 (36.8)
2020, *n* (%)	32 (57.1)
2021, *n* (%)	10 (58.8)

ACS, acute coronary syndrome; CBF, cerebral blood flow; CPR, cardiopulomoary resuscitation; NYHA, New York Heart Association.

**Table 2:** Prognostication modalities and trend over time Modalities were only rated as “performed” if performance was in accordance with the German Society of Neurology guidelines [13] regarding time point and exclusion of confounders. Trends over time were analysed by logistic regression.

Modality	2016 (*n* = 23)	2017 (*n* = 34)	2018 (*n* = 59)	2019 (*n* = 38)	2020 (*n* = 56)	2021 (*n* = 17)	Total (*n*, %)	*p*-Value	Exp (B)	95%CI
cCT, *n* (%)	19 (82.6)	27 (79.4)	41 (69.5)	33 (86.8)	46 (82.1)	11 (64.7)	177 (78.0)	0.88	0.98	0.79–1.22
Pupillary light reflex, *n* (%)	18 (78.3)	29 (85.3)	45 (76.3)	29 (76.3)	36 (64.3)	15 (88.2)	172 (75.8)	0.26	0.88	0.72–1.09
EEG, *n* (%)	19 (82.6)	29 (85.3)	44 (74.6)	24 (63.2)	41 (73.2)	12 (70.6)	169 (74.4)	0.12	0.84	0.68–1.04
SSEP, *n* (%)	17 (73.9)	25 (73.5)	37 (62.7)	25 (65.8)	38 (67.9)	14 (82.4)	156 (68.7)	0.93	1.01	0.83–1.22
NSE at 24 h, *n* (%)	12 (52.2)	17 (50.0)	38 (64.4)	30 (78.9)	50 (89.3)	16 (94.1)	163 (71.8)	<0.001	1.76	1.40–2.22
NSE at 72 h, *n* (%)	10 (43.5)	16 (47.1)	30 (50.8)	25 (65.8)	34 (30.7)	10 (58.8)	125 (55.1)	0.062	1.19	0.99–1.43

SSEP, somatosensory evoked potentials; EEG, electroencephalography; cCT cranial computed tomography; NSE, neuron-specific enolase; Exp (B), exponent b; CI, confidence interval.

**Table 3:** Neurological prognostication and in-hospital outcome prognostic categories were determined according to the German Society of Neurology guideline [13]. Logistic regression was performed to evaluate the correlation of survival status and advancing years.

Prognostic Category	2016 (*n* = 23)	2017 (*n* = 34)	2018 (*n* = 59)	2019 (*n* = 38)	2020 (*n* = 56)	2021 (*n* = 17)	Total	WLST (Yes/No)	CPC 4	CPC < 4
	Death (Survival to discharge)	Death (Survival to discharge)	Death (Survival to discharge)	Death (Survival to discharge)	Death (Survival to discharge)	Death (Survival to discharge)	Death (Survival to discharge)			
Poor	9 (0)	15 (0)	29 (0)	19 (0)	33 (0)	11 (0)	116 (0)	113 /3	0	0
Very likely poor	2 (2)	6 (1)	13 (0)	5 (1)	11 (1)	2 (0)	39 (5)	38 /6	4	1
Indeterminate	5 (3)	3 (7)	4 (8)	5 (5)	3 (5)	1 (3)	21 (31)	19 /33	8	23
Good	0 (0)	0 (1)	0 (0)	0 (1)	0 (1)	0 (0)	0 (3)	0 /3	0	3
Missing	2	1	5	2	2	0	12	7/ 5		
								***p*-value**	**Exp (B)**	**95%CI**
Death (Survival to discharge), %	78.3 (21.7)	73.5 (26.5)	86.4 (13.6)	81.6 (18.4)	87.5 (12.5)	82.4 (17.6)	82.8 (17.2)	0.24	1.16	0.91–1.45

WLST, withdrawal of life supporting therapy; CPC, cerebral performance category; Exp (B), exponent b; CI, confidence interval.

## Data Availability

The data presented in this study are available upon request from the corresponding author. The data are not publicly available due to data protection guidelines.
